# Use of Milk Fat/Cellulose Ether Emulsions in Spreadable Creams and the Effect of In Vitro Digestion on Texture and Fat Digestibility

**DOI:** 10.3390/foods9060796

**Published:** 2020-06-17

**Authors:** Maria Espert, Teresa Sanz, Ana Salvador

**Affiliations:** Instituto de Agroquímica y Tecnología de Alimentos (CSIC), Avda. Agustín Escardino 7, 46980 Paterna, Spain; mespert@iata.csic.es (M.E.); tesanz@iata.csic.es (T.S.)

**Keywords:** hydrocolloids, milk fat, spreadable creams, spreadability, fat release

## Abstract

This study investigated the texture properties and fat digestibility of new spreadable chocolate creams formulated with an emulsion composed of milk fat and a cellulose ether as a fat source. The spreadability was analysed at 20 °C and compared with a commercial spreadable cream formulated with palm fat. Structural changes in the creams after the in vitro oral and gastric digestion stages were evaluated; lipid digestibility was determined by titration with NaOH during intestinal digestion. Spreadability tests showed the spreads were similar. After oral digestion, the commercial spread showed an increase in extrusion force because of flocculation induced by saliva, an effect not observed in spreads with cellulose ether. Digestibility determination showed lower values for the reformulated spreads. Therefore, milk fat-cellulose ether based emulsions offer an alternative to achieve reformulated spreadable creams, with physical properties similar to those of commercial products but providing reduced fat content and lower lipid digestibility, without compromising the quality of the final product.

## 1. Introduction

Solid fats (fats high in saturated fatty acids) are major components in several food products used daily including spreads, creams, chocolates, ice-creams, and various bakery products [1]. These saturated fats have a positive influence on the flavour, taste, texture, and overall acceptability of the food products. However, food safety authorities have pursued the reduction of fat content, leading to multiple reformulation strategies [2] aimed at providing acceptable organoleptic and physical properties of the full-fat counterparts without compromising the eating quality.

Chocolate spread is a good candidate for reformulation, as they are high in fat (higher than 40 g/100 g), mostly saturated (palm oil, coconut oil, and cocoa butter), which provides the inherent product texture, mouthfeel, and flavour [3]. However, the partial substitution of saturated fat with unsaturated oils has not proved suitable, as it compromises the final quality of the confectionary product [4]. An approach to minimize the impact of fat reduction is the use of edible emulsions. Oil-in-water emulsions are common in food products, especially in products considered high in fat [2]. To reduce fat content, the addition of non-fat ingredients (e.g., hydrocolloids), that increase the viscosity of the aqueous phase in a solid fat-water emulsion, achieve fat reduction in a food matrix but maintain the intrinsic fat properties. A possible drawback in the use of containing water emulsions is the moisture they provide; as is well-known, edible spreads containing water are more vulnerable to microbiological deterioration. Notwithstanding, this can be controlled by ensuring a stabilization of the aqueous phase, that can be achieved by increasing the viscosity of the moisture droplets through the use of stabilizers or thickeners [5,6,7].

A recent study [8] has shown that anhydrous milk fat emulsions with added cellulose ethers proved a viable alternative in reduced fat spreads with improved physicochemical characteristics than conventional butter, resulting in a spreadable product at a wide temperature range (5 °C and 20 °C). Authors reported that cellulose ethers in a concentrated oil emulsion has a significant impact on lipid digestibility within the digestive tract [9], with only a low percentage of the contained fat being bio-accessible to the body and the rest therefore eliminated without digestion. This hypothesis would be key in solid fat emulsions, although these formulated emulsions would not result in a lipid profile improvement, the decrease in digestibility would represent an interesting approach to fat reduction.

The objective of this study was to evaluate the potential fat reduction in chocolate spreads by using anhydrous milk fat-based emulsions, with different cellulose ethers were used as carbohydrate-based stabilizers. The mechanical properties of the chocolate spread matrix before and after in vitro digestion were evaluated, as well as the digestibility of the fat.

## 2. Materials and Methods

### 2.1. Emulsion Preparation

Different oil-in-water (o/w) emulsions were stabilized using three cellulose ethers with different chemical substitution: methylcellulose Methocel™ A4M (30.0% methoxyl), methylcellulose Methocel™ MX (>30.0% methoxyl), and hydroxypropyl methylcellulose Methocel™ F4M (28% methoxyl, 5% hydroxypropyl, molecular weight of 9500 Da). All of them were provided by The Dow Chemical Company (Midland, MI, USA).

Emulsions were prepared using these proportions: 47% (w/w) milk fat (Pure Butter Ghee, East End Foods PLC), 2% (w/w) hydrocolloid, and 51% (w/w) water. Milk fat was melted in a water bath at 65 °C and then left to cool to a temperature of 40–45 °C. The cellulose ether was dispersed in milk fat using a Heidolph stirrer (Heidolph Instruments GmbH & Co. KG, Schwabach, Germany) at 280 rpm for 5 min. The mixture was hydrated by gradually adding 20 °C water with continuous stirring. Finally, the emulsion was homogenised using an Ultra-turrax T18 homogeniser (IKA, Staufen im Breisgau, Germany) at 6500 rpm for 1 min then 17,500 rpm for 1 min.

### 2.2. Spreadable Creams Preparation

Emulsion based cocoa creams comprised water (19%), sugar (Disem, Madrid, Spain) (15%), skimmed milk powder (SMP) (Central Lechera Asturiana, Granda, Asturias, Spain) (5%), cocoa powder (Chocolates Valor S.A., Alicante, Spain) (11%), and cellulose-milk fat based emulsion (50%). A food processor (TM31 Thermomix, Vorwrek, Wuppertal, Germany) was used to mix the ingredients. The sugar, SMP, cocoa powder, and mineral water were mixed at 90 °C for 6 min at speed 2. The mixture was cooled at room temperature. The hydrocolloid emulsion was added and mixed in the food processor for 6 min at speed 2 without a temperature selection to obtain the spreadable cream.

A commercial cocoa spread (Nutella, Ferrero Ibérica, S.A., Barcelona, Spain) was the control. According to the label declaration, the composition of this product is sugar (53%), palm fat (30.9%), hazelnuts (13%), SMP (8.7%), fat-reduced cocoa powder (7.4%), soya lecithin, and vanillin. The total fat content is 30.9% and the saturated fat content is 10.6%. In the formulated spreadable creams, the total fat content was 23.5% and the saturated fat content is 15.6%. All the spreads were stored at 20 °C for 24 h before analysis.

### 2.3. In Vitro Digestion

In vitro digestion was performed following the adapted methodology previously used by Espert et al. [10]. This model simulates the different digestion phases (oral, gastric, and small intestine digestion) by applying specific physiologically conditions (digestive fluids, pH, temperature and specific time in each step).

### 2.4. Fat Digestibility

Free fatty acids (FFA) released at the end of in vitro digestion were calculated. The intestinal digestion was carried out over 2 h whilst maintaining the pH at 7.0 by addition of 0.1N NaOH solution (Panreac Química S.L.U., Spain) using a pH-stat automatic titration (Mettler Toledo, Barcelona, Spain). The volume of NaOH added to neutralize the samples was used to calculate the FFA release according to Li and McClements [11].

### 2.5. Textural Properties

TA-XT plus Texture Analyser equipped with the Texture Exponent software (Stable Microsystems, Godalming, UK) was used to determinate the texture properties of the samples.

Spreadability of samples was measured using a TTC spreadability rig (HDP/SR) (Stable Microsystems, Godalming, UK) attachment. Samples were filled into a female cone (90°) with special attention to avoid bubbles formation. Samples were penetrated to 22 mm using a corresponding male cone (90°) attachment at a speed of 1 mm/s. Force expressed in Newtons and area under the curve (AUC; N·s), as a measure of spreadability, were recorded. Measurements were performed in triplicate.

A back extrusion test was conducted using a bucket of 50 mm diameter and 75 mm height with a compression probe of 49 mm diameter. The samples were extruded by 15 mm, at a speed of 1 mm/s. From the force time profiles, the AUC (N·s), a measure of consistency, was recorded. Measurements were performed in triplicate.

### 2.6. Statistical Analysis

Levene’s test was used to assess homogeneity of variances across groups. Analysis of variance (ANOVA) was performed on the data using XLSTAT statistical software (version 5 May 2014, Microsoft Excel^®^, Barcelona, Spain). The means were compared using the Tukey test and the statistical significance was determined at *p* < 0.05.

## 3. Results and Discussion

### 3.1. Appearance of Cocoa Spreads with Milk Fat Emulsion

Cocoa spreads designed using milk fat/cellulose ether emulsions as a fat source contain 23.5% total fat, a significant decrease from other fat sources such as palm fat, vegetable shortenings and butter which containing 80% fat. In this case, milk fat was used instead of olive or sunflower oil (low in saturated fatty acids), because the texture of fat spreads formulated with liquid oils do not have the plastic properties of solid fats, and their texture is fluid. In addition, during preliminary tests, milk fat was found to be the most effective (compared to liquid oil) in minimizing the gummy taste provided by cellulose ethers.

**Figure 1:**
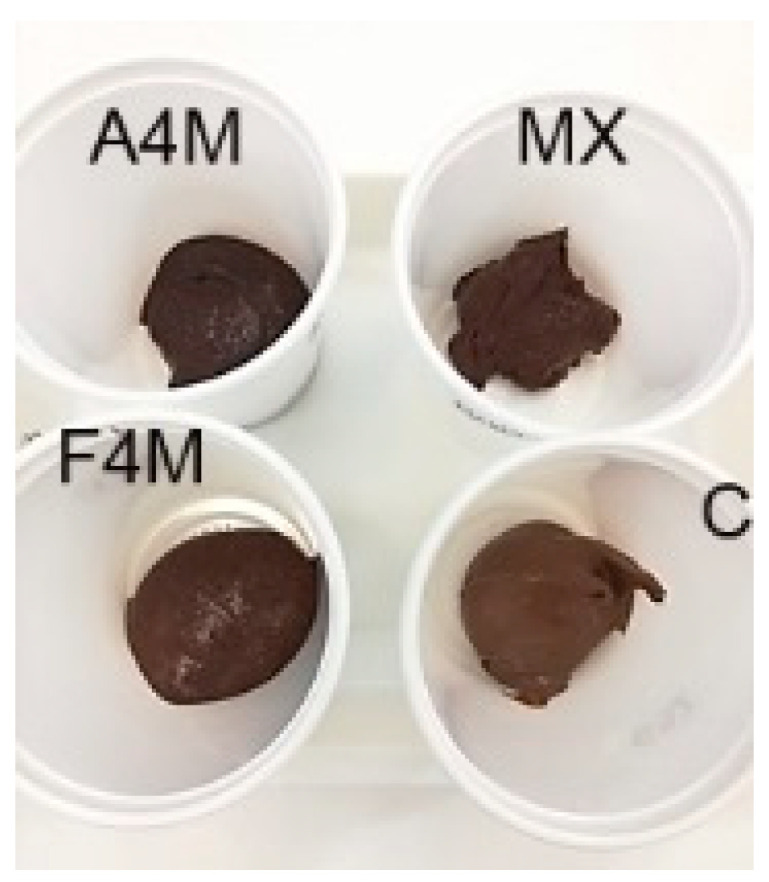
Appearance of formulated cellulose ethers spreads compared to a commercial control (A4M: methycellulose Methocel A4M; MX: methycellulose Methocel MX; F4M: hydroxypropylmethycellulose Methocel F4M; C: commercial cocoa spread).

The visual appearance of the cocoa spreads with milk fat/cellulose ether emulsions is presented in Figure 1. The three spreads presented a spreadable texture like the commercial control cream. The MX spread was the least similar to the control, presenting a lumpy and aerated texture, similar to that of a mousse.

### 3.2. Texture

#### 3.2.1. Spreadability Test

**Figure 2:**
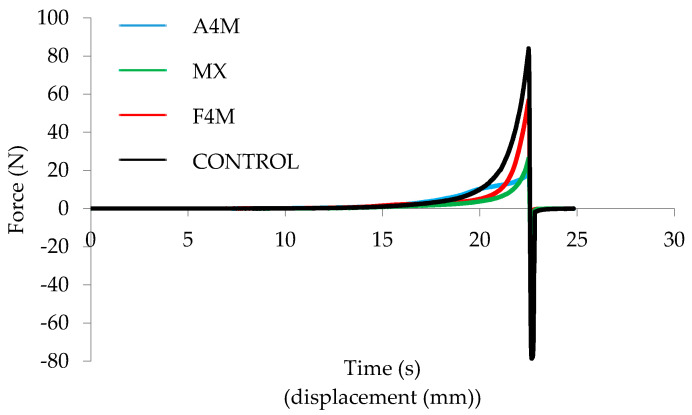
Spreadability curves of the different studied spreads.

The chocolate spreads on the market are characterised by their plastic and spreadable character; here, reduced-fat formulated spreads were compared with the commercial control cream, with respect to the spreading capacity. Figure 2 shows the spreadability profiles of the samples at 20 °C. The control cream showed a higher maximum force than the creams with emulsion, although the cream with F4M exhibited a similar profile.

To better appreciate the significant differences between the samples, Table 1 shows the values of maximum force and AUC calculated from the curves data. The maximum force and AUC were significantly higher in the control cream, while F4M spread shows significantly higher force values than the MX and A4M spreads. MX cream was the one with the lowest peak force and AUC.

This difference could be associated with the presence of lumps and the aerated texture of this sample. The different texture provided by the MX spread could be explained because of the different texture also shown by the initial MX emulsion in a previous study [8]. This study showed that compared to other cellulose ether emulsions, MX showed lower firmness and lower viscoelastic functions, fact that was attributed to the lowest level of crystallization observed in the microstructure, smaller than the other methylcellulose cellulose (A4M) ether spreads. The presence of smaller crystals forms weaker links and these result in a decrease of viscoelasticity [12]. The F4M emulsion showed the highest values of G’ and the closest behavior to butter, while MX emulsion showed the lowest viscoelasticity. In the F4M emulsion a more dense crystalline structure is observed [8]. These results explain that the texture of the F4M spread at 20 °C is also harder and close to control spread, so this spread has an optimal behavior and could be an alternative to commercial full-fat spread.

#### 3.2.2. Back Extrusion Test

**Figure 3:**
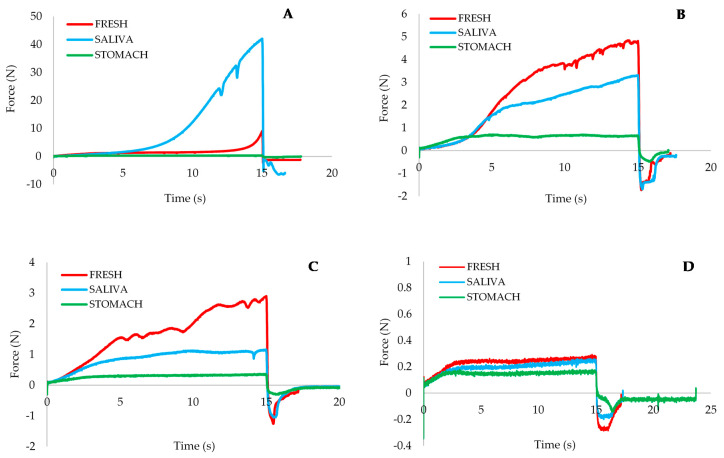
Extrusion profiles of the studied spread and after oral and gastric in vitro digestion. ((**A**) control; (**B**) A4M; (**C**) F4M; (**D**) MX).

To evaluate changes in sample texture caused by in vitro digestion, a back extrusion test was conducted at 37 °C (simulating body temperature). Representative extrusion curves of the different creams before and after oral and gastric in vitro digestion are presented in Figure 3.

Formulated spreads showed similar behavior during digestion. A decrease in extrusion force was observed after in vitro digestion in the mouth but was much greater after stomach digestion.

The behavior of the control sample after oral digestion differed completely from the spreads with emulsion. The control sample showed a notable increase in extrusion force after oral digestion. In contact with saliva, the commercial spread became consistent, heterogeneous, and sticky; associated with a saliva-induced flocculation effect. As is known, saliva induces almost instantaneous flocculation of emulsions [13,14]. Droplet aggregation in contact with saliva was also found in fish oils emulsified with lecithin, attributed to depletion flocculation induced by non-adsorbed mucin, or electrostatic screening caused by inorganic salts [15]. This difference between commercial and cellulose-based spreads would explain the barrier effect exerted by the cellulose against the action of saliva. A lack of effect of saliva on the structure of sunflower oil/cellulose emulsions was also observed; the microstructure, particle size distribution and viscoelastic properties were not affected [9,16]. Custard desserts prepared with HPMC or CMC were also not affected by mixing with saliva [12,17].

This difference in the saliva effect between control and cellulose-based spreads would lead to a different sensory perception in the mouth. The control cream would show an increase in hardness/consistency perceived during mixing with saliva, not perceptible in the reformulated spreads.

After the gastric phase all the samples, including the control, were more fluid, showing a decrease in the extrusion force as their structure was altered due to the presence of enzymes, the change in pH and temperature and the dilution that occurs at this stage.

The AUC gives an objective evaluation of the spreads’ consistency. Table 2 shows the mean values of this parameter for each sample at each digestion stage. A significant increase (*p* < 0.05) was observed in the control sample after incubation with saliva, which confirms the aforementioned hypothesis.

### 3.3. Determination of FFA after In Vitro Digestion

**Figure 4:**
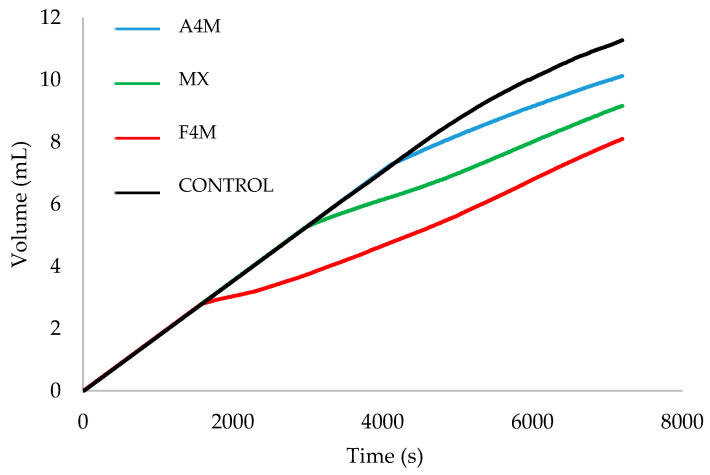
Volume of NaOH consumed during intestinal digestion of the studied spreads.

Figure 4 shows the volume of NaOH consumed by each spread sample as a function of time, during incubation with intestinal fluids. A higher consumption of NaOH indicates an increased production of FFA during incubation, which would result in greater fat digestibility.

During the first 30 min of incubation, a rapid increase in the consumption of NaOH is observed, neutralizing the FFA generated during the fat digestion, with no differences were between samples. However, this increase was followed by a more gradual release for the last digestion phase. This phenomenon may be due to the fact that the lipolysis products accumulated at the droplet interface, which restricted the access of the enzym complex to the triacylglycerides.

After 30 min of intestinal incubation differences were observed between the samples, with a decrease of the curves’ gradients for the cellulose ether spreads. The F4M spread had the lowest NaOH consumption when the trial ended, followed by the MX and A4M spreads. This lower NaOH consumption indicates a lower digestibility in the emulsion-based spreads than the control spread. Therefore, cellulose ethers reduce the effectiveness of bile salts and digestive enzymes in fat digestion.

To compare the differences between samples, Table 3 shows the release of FFA (%) after in vitro digestion of each spread.

The F4M spread presented significantly lower values than the control spread, and no significant differences with the other cellulose ether spreads. Although no significant differences were observed between the cellulose ether spreads, the fat release profile during intestinal digestion was lower in case of F4M cream. This finding can be attributed to its greater hardness and the different fat crystals morphology in the initial emulsion, which consists of a dense crystalline structure with larger crystals compared to A4M and MX emulsions [8]. Previous studies confirm that the polymorphic organization of the lipids in crystals may result less accessible to the enzymes than the liquid state [18]. At the same time, larger fat crystals may have greater spatial resistance to prevent the adsorption of the enzymatic complex [19].

## 4. Conclusions

Milk fat-cellulose ether based emulsions represent a feasible alternative for the elaboration of reformulated spreads with reduced fat content which maintain their plastic properties. In this study, hydroxypropyl methylcellulose (F4M) provided the most consistent textured spread, very similar to commercial spreads. Besides, these emulsion-based creams present a lower digestibility, which is associated with the barrier effect provided by cellulose of the emulsion against fat digestion. The presence of cellulose ethers reduces the effectiveness of saliva, bile salts and digestive enzymes during in vitro digestion, so the bioavailability of fat is lower than in commercial spreads. Although from a nutritional point of view the milk fat used has a high percentage of saturated fatty acids, cellulose based emulsions contribute to the spreads nutritional improvement, since the emulsion has a lower initial fat content and reduces digestibility in the final product. Furthermore, cellulose ether emulsion formulated with milk fat helps to accomplish the characteristic plasticity of saturated fats that cannot be achieved with liquid oils, thus providing consistency and spreading properties like those of a commercial spread.

## Figures and Tables

**Table 1:** Textural parameters (mean ± standard deviation) from penetration curves of the different spreads at 20 °C.

Spreads	Max. Force (N)	AUC (N·s)
Control	85.38 ^a^ ± 2.04	109.55 ^a^ ± 13.00
A4M	19.84 ^c^ ± 1.76	56.73 ^b^ ± 1.64
F4M	66.61 ^b^ ± 0.95	57.41 ^b^ ± 0.84
MX	23.26 ^c^ ± 4.27	37.54 ^c^ ± 5.40

AUC: Area under the curve. ^abc^ Values with the same letter for each parameter are not statistically different (*p* < 0.05).

**Table 2:** Extrusion parameter (AUC) (mean ± standard deviation) of the fresh spreads and after in vitro digestion.

Creams	Digestion Step	AUC (N·s)
Control	Fresh	27.42 ^a^ ± 4.22
	Saliva	151.93 ^b^ ± 36.25
	Stomach	3.78 ^a^ ± 0.26
A4M	Fresh	38.24 ^a^ ± 1.73
	Saliva	25.37 ^b^ ± 2.81
	Stomach	9.63 ^c^ ± 1.09
F4M	Fresh	23.27 ^a^ ± 2.25
	Saliva	13.95 ^b^ ± 1.45
	Stomach	4.18 ^c^ ± 0.24
MX	Fresh	3.57 ^a^ ± 0.15
	Saliva	2.87 ^b^ ± 0.16
	Stomach	2.30 ^c^ ± 0.13

AUC: Area under the curve. ^abc^ Values with the same letter for each spread are not statistically different (*p* < 0.05).

**Table 3:** FFA release at the end of the in vitro digestion expressed as a mean ± standard deviation.

Spreads	FFA (%)
Control	62.61 ^a^ ± 0.82
A4M	61.40 ^a,b^ ± 0.26
MX	52.10 ^a,b^ ± 0.27
F4M	46.25 ^b^ ± 0.20

^ab^ Values with the same letter are not statistically different (*p* < 0.05).
